# A Novel Approach for Conservative Management of Placenta Accreta Spectrum Disorder Cases: Experience of a Single Surgeon: PAS Disorders and Conservative Management

**DOI:** 10.1155/2024/9910316

**Published:** 2024-06-12

**Authors:** Ahmet Yalınkaya, Süleyman Cemil Oğlak

**Affiliations:** ^1^ Department of Obstetrics and Gynecology Dicle University Faculty of Medicine, Diyarbakır, Türkiye; ^2^ Department of Obstetrics and Gynecology Health Sciences University Gazi Yaşargil Training and Research Hospital, Diyarbakır, Türkiye

**Keywords:** conservative surgical technique, massive hemorrhage, placenta accreta spectrum

## Abstract

**Background:** This study is aimed at evaluating the conservative surgical treatment of patients with placenta accreta spectrum (PAS) disorder and at presenting the experience of a single surgeon.

**Materials and Methods:** This retrospective study included 245 patients with placenta previa accompanied by PAS disorders operated at a university hospital between June 2013 and December 2023. The diagnosis of PAS was made by a single perinatologist using a combination of transvaginal and transabdominal ultrasonography. All patients were operated with conservative surgical technique by the same surgeon. The demographic and clinical characteristics of the patients, the anesthesia and incision types used, and the details of the surgical technique were evaluated.

**Results:** Of the patients, 165 were operated on at the scheduled time, 80 were operated on under emergency conditions, and 232 (94.69%) of them were operated on under spinal anesthesia. All patients were operated on with a Pfannenstiel incision followed by a transverse incision to the upper border of the placenta to enter into the uterus. An average of 0.52 units of red blood cells per patient was transfused to all patients. Spontaneous intra-abdominal bleeding developed in five patients, and surgical complications occurred in eight patients. No cesarean hysterectomy was performed, and no maternal mortality was detected in any of the cases. The mean time duration of surgery was 54.44 ± 11.37 (30–90) min, and the mean length of hospital stay was 1.71 ± 1.30 (1–9) days.

**Conclusions:** We recommend this procedure as a novel technique and a robust and safe alternative to peripartum hysterectomy and other conservative surgical management procedures for cases with complete PP accompanied with PAS. This technique preserves the uterus as well as reduces blood loss, and transfusion requirement, and thus maternal morbidity and mortality in PAS cases.

## 1. Introduction

Placenta accreta spectrum (PAS) disorders can cause life-threatening massive bleeding during pregnancy. PAS disorders refer to all pathological placental adhesion anomalies including placenta accreta, increta, and percreta. The two latter forms go to the deeper layers of the uterus or beyond the uterine serosa, respectively. The clinical result of abnormal placentation is the failure of placental separation leading to massive postpartum hemorrhage with a significant increase in maternal morbidity and mortality [1, 2]. Usually, PAS disorders are diagnosed by ultrasound (US) examination. Many US findings have been described in patients with PAS disorders, such as irregular vascular spaces, loss of normal hypoechoic retroplacental zone, thinning or absent myometrium, protrusion of the placenta into the bladder, increased vascularity of the uterine serosa-bladder interface, and turbulent blood flow through the lacuna on Doppler US [3].

The worldwide incidence of PAS is rapidly increasing, following the rising trend of cesarean delivery, with an estimated range from 1 to 3 per 1000 births. A systematic review of near-miss cases of PAS disorders indicated that the invasion in the inferior part of the lower uterine segment, posterior bladder, and parametria was associated with a high risk of maternal morbidity [4]. Patients with PAS disorders are associated with an increased rate of maternal morbidity and mortality, including massive blood transfusion, cystotomy, postpartum infection, peripartum hysterectomy, prolonged hospital stay, intensive care unit (ICU) admission, and even death [5–8]. PAS disorders are significant life-threatening issues due to the increased incidence, morbidity, and mortality, and it is called “obstetrician's nightmare” among obstetricians [9, 10].

PAS disorders can cause many obstetric severe problems in the peripartum period, such as disseminated intravascular coagulopathy (DIC), the need for hysterectomy, and surgical injuries to the ureter, bladder, intestine, or neurovascular structures, as well as acute respiratory distress syndrome, blood transfusion reactions, electrolyte imbalance, kidney damage, and multiorgan failure. Also, the average blood loss at delivery in patients with placenta accreta is 3.000–5.000 mL; 90% of the cases need blood transfusion and 40% more than 10 units of packed red blood cells (RBCs). It has been reported that maternal deaths in PAS cases varied between 1% and 7% worldwide [11–14].

The standard surgical treatment recommended for PAS is usually a cesarean hysterectomy, but the loss of the uterus leaves women infertile as well as reduces the woman's self-esteem and societal status in some countries. Therefore, over the past two decades, many conservative approaches have been reported to preserve fertility in these patients [15–19]. International Federation of Gynecology and Obstetrics (FIGO) guidelines reported four different primary conservative treatment modalities for PAS disorders: the extirpative technique, leaving the placenta in situ or the expectant approach, one-step conservative surgery, and the Triple-P procedure [20]. In addition to these procedures, interventional radiology procedures are also applied. These methods have been used alone or in combination [21]. Although many modified conservative methods have been tried in the last two decades and these methods have significantly reduced maternal morbidity and mortality, they are still insufficient, and unfortunately, PAS disorders continue to be challenging for obstetricians worldwide [22].

In this study, we aimed to present a conservative surgical technique in patients with PAS disorders and to contribute to the literature with a new perspective.

## 2. Materials and Methods

### 2.1. Study Design and Participants

**Figure 1:**
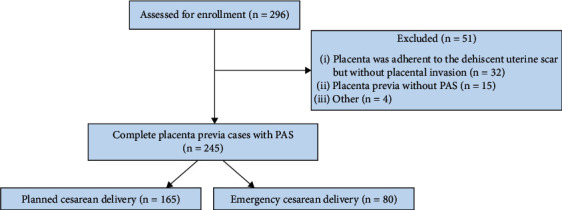
Flowchart of the study.

This retrospective study was designed at Dicle University Faculty of Medicine Hospital in Diyarbakır, Turkey, between June 2013 and December 2023, and included 245 patients with placenta previa (PP) complicated with PAS disorders who were operated on by a single surgeon (A.Y.) using a conservative surgical technique with his multidisciplinary team. The study project was approved by the Institutional Ethics Committee of Dicle University Faculty of Medicine (2024/101). This study includes only patients who were antenatally diagnosed with complete PP accompanied with placental invasion to the previous cesarean scar, and this diagnosis was surgically confirmed at the delivery time. The study included only the PAS cases managed by A.Y. All pregnant women were selected based on the preoperative US findings and surgically confirmed. PP cases without PAS and cases who were unwilling to receive uterus-preserving surgery were excluded. PAS cases with suspected invasion below the bladder trigone and lower parametrial invasion were also excluded due to the high failure risk of the conservative surgery (Figure 1).

According to US guidelines, an experienced perinatologist (A.Y.) using a combination of transabdominal and transvaginal GE Voluson E8 US devices at least once during the prenatal period confirmed the diagnosis of all patients. The diagnosis of PP was usually performed during the second-trimester fetal anomaly scan. After this diagnosis, patients were assessed by transabdominal and transvaginal sonography regarding placental localization in the third trimester by an experienced maternal-fetal medicine specialist (A.Y.). Also, the diagnosis was confirmed at the time the pregnant patient was admitted to the hospital for delivery or hemorrhage and finally during the surgery. All cases were operated, and the diagnosis of all cases was confirmed by the same surgeon (A.Y.).

PP was defined as the placental tissue overlying the internal cervical os. Prenatal diagnosis of PAS was made according to the following US findings: loss of the clear zone, abnormal placental lacunae, bladder wall interruption, and uterovesical hypervascularity [23]. Loss of the clear zone was described as the loss or irregularity of the hypoechoic plane in the myometrium underneath the placental bed. Abnormal placental lacuna was identified as the existence of numerous lacunae, frequently including turbulent flow detectable on grayscale or color Doppler US. Bladder wall interruption was defined as the loss or interruption of the bright bladder wall (hyperechoic band or line between bladder lumen and uterine serosa). Uterovesical hypervascularity was described as the striking amount of color Doppler signal detected between the myometrium and posterior wall of the bladder, including vessels seeming to extend from the placenta, across myometrium, and beyond serosa into the bladder or other organs, frequently running perpendicular to the endometrium. Surgical diagnosis of PAS disorder was confirmed based on the FIGO guidelines [24]. The placental invasion was not confirmed by histopathological examination because tissue resection or hysterectomy was not performed in any of the cases.

According to our clinical protocol, patients with persistent PP accompanied by PAS in the third trimester were scheduled to undergo a cesarean delivery between 37^0/7^ and 38^0/7^ weeks of pregnancy. All patients and their relatives were informed about the disease and were hospitalized or stayed close to the healthcare facility as much as possible in the last trimester. All multidisciplinary team members were informed of the scheduled day to ensure the availability of staff and the booking of the operating room.

Details of the new surgical technique, type of anesthesia, pre- and postoperative mean hemoglobin (Hb) values, estimated blood loss (EBL), antenatal spontaneous complications, and postpartum surgical complications were assessed separately. Total blood transfusion, time duration of surgery, postoperative ICU need, and length of hospital stay were evaluated. The consent and approval of all patients for the operation were obtained. While the patients were discharged, their epicrises were added to the files of the previous PAS patients and the data of all patients were recorded on a special computer. The duration of surgery was defined as the time interval from skin incision to closure in minutes.

EBL was calculated with the following formula: ΔHb (the difference between postoperative and preoperative Hb value) = 1.001 × EBL (liters) + 0.441 × intravenous fluids (liters) + 2.334 [25]. The amount of intravenous fluids given was obtained from operative procedure notes and the anesthesia operative notes.

### 2.2. Details of Surgical Technique

All cases were delivered by a single surgeon (A.Y.) who was trained and experienced more than 20 years in surgery for PP accompanied with PAS disorders.

#### 2.2.1. Opening of the Abdominal Wall

At least 30 min, but no greater than 60 min before the skin incision, 2 g of a first-generation cephalosporin was administered in all cases. If there is no medical contraindication, after abdominal and vaginal povidone-iodine antisepsis, a cesarean section is performed under spinal anesthesia with a Pfannenstiel incision. Spinal anesthesia allows performing adhesiolysis carefully and gently if there are adhesions between the abdominal wall and subperitoneal internal/pelvic organs. After entering the abdominal cavity, a transverse uterine incision was performed to enter the uterus and deliver the fetus. The uterine incision is created transversely, just above the upper border of the placenta, ensuring that the myometrium's natural thickness is preserved. When the border of the placenta extends higher, the incision is placed where the myometrial tissue is thicker if possible.

#### 2.2.2. Delivery and Hemostasis

**Figure 2:**
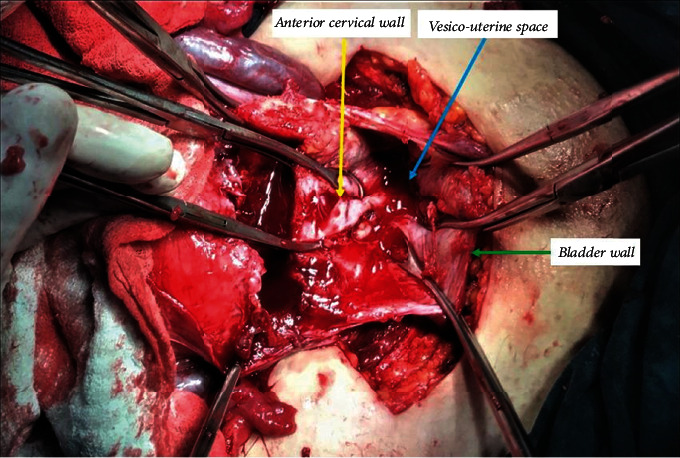
Intraoperative appearance of the gap formed after removal of the placenta percreta from the vesicouterine space.

The neonate was grasped and then delivered smoothly through the uterine incision. Following the fetus extraction, the umbilical cord was clamped without separation of the placenta. The uterus is exteriorized together with the placenta. Intravenous oxytocin 10 IU infusion was administered in all patients following the delivery of the fetus. The surgeon grasps and squeezes the lower uterine segment circularly from the back, thereby mechanically preventing blood loss (hand tourniquet), and then tries to separate the whole placenta by squeezing it with the other hand. After the separation of the placenta, all bleeding areas are clamped with several curved ovarian forceps. After the mechanical hemostasis of the lower uterine segment has been achieved, the remaining small amounts of placental fragments are removed by instruments with an aim for complete removal (Figure 2).

#### 2.2.3. Repair and Closure of the Uterine Wall

**Figure 3:**
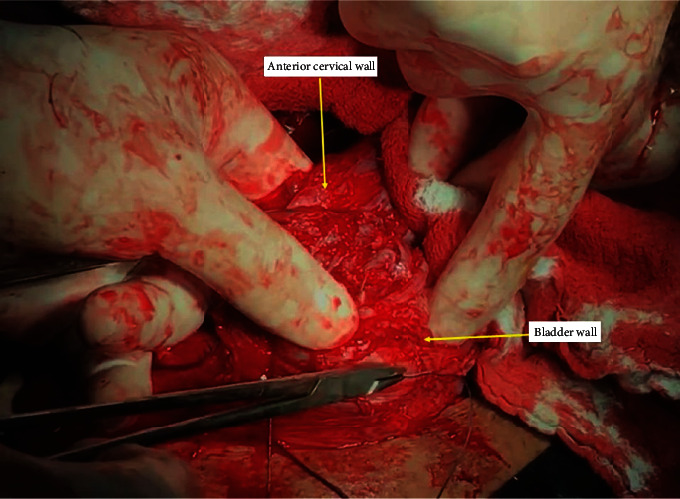
Closing the vesicouterine space with the guiding of the fingers and creating the anterior lower edge of the incision.

**Figure 4:**
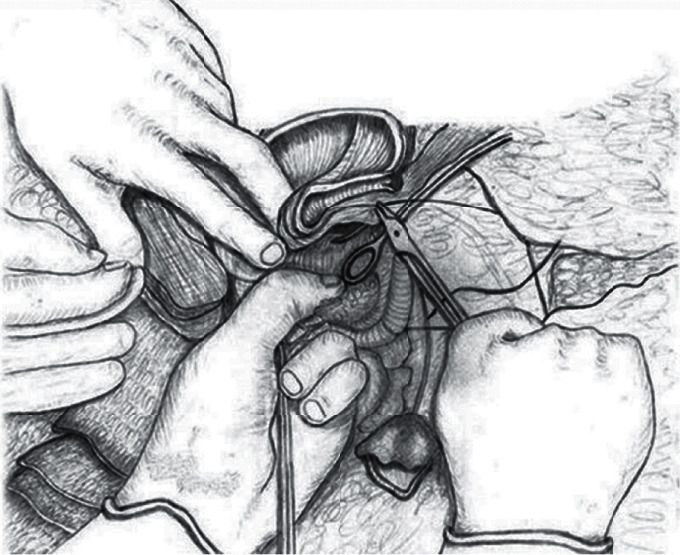
The schematic appearance of suturing the vesicouterine space by guiding the fingers (this drawing was drawn by Prof. Dr. Vatan Kavak, who is an anatomist artist).

Following the achievement of the mechanical hemostasis in the lower uterine segment, sutures are placed on both the left and right corners of the uterine incision. The clamps are removed sequentially, and the vesicouterine interface and all spaces are sutured with superficial stitches under the guidance of the surgeon's fingers. These superficial continuous sutures are not very deep but are placed around the tissue surrounding the vessel to put the squeeze on it and obtain hemostasis. Particular care should be taken to avoid the ureter or bladder injury during this procedure. Then, starting the suturing from the left or right corner of the uterus, the lower edge of the incision is created with continuous locked and unlocked sutures (Figure 3). The surgeon should insert the index finger into the cervical canal for guidance while creating the anterior lower edge of the incision and feel the place where the needle passes with his fingers to avoid passing the suture from the cervical canal (Figure 4). If hemostasis is not achieved in the underlying placental bed and surrounding structures, multiple hemostatic sutures are directly placed to cease the bleeding. After creating the lower edge of the uterine incision, the uterine incision was then closed by double layers and continuously locked and unlocked sutures (Video 1 (https://drive.google.com/file/d/1zb3-gaK0_ZQDAsl9hkSHM0iNFOnZre3X/view?usp=sharing)).

#### 2.2.4. Compression Sutures on the Incision

**Figure 5:**
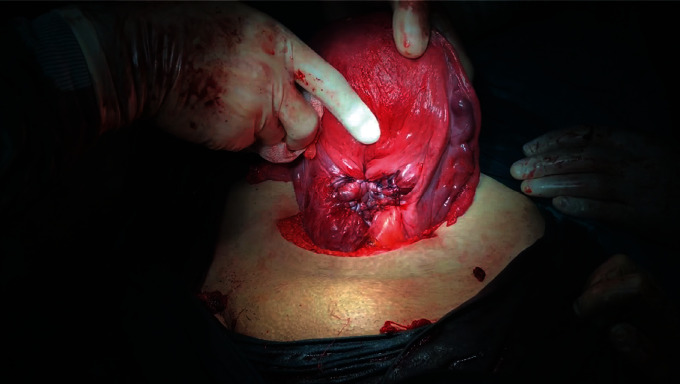
The appearance of the uterine incision after inward compression suture.

The lateral sutures are passed from the opposite side and pulled towards each other, the incision is bent inward, and the sutures are tied. These sutures provide the concavity of the middle of the incision and allow the inner endometrial surfaces to contact each other, thereby compressing the endometrial surface and preventing superficial blood leaks, and may reduce the likelihood of developing a subsequent isthmocele (Figure 5 and Video 2 (https://drive.google.com/file/d/1fJznN5MoB_TAtwEhnBWoF5gp8kvy-tz_/view?usp=sharing)).

### 2.3. Statistical Analysis

IBM SPSS version 21.0 software (SPSS Inc., Chicago, IL, USA) statistical program was used for analyzing the research data. General descriptive statistics were presented as counts and percentages for categorical variables, and mean ± standard deviation (minimum–maximum) or median (IQR1-3) for continuous variables. The Kolmogorov–Smirnov test was performed to determine the normality distribution. Continuous variables were compared using the Student *t*-test or Mann–Whitney *U* test among the groups. The chi-square test was used to detect differences in categorical data by groups. Statistical tests were considered significant at *p* value < 0.05.

## 3. Results

Maternal demographic characteristics and clinical findings of all patients (*n* = 245) were evaluated in detail according to planned and emergency patients (Table 1). The mean maternal age of the study cohort is 32.84 ± 5.02 years, and the mean previous number of cesarean sections is 3.20 ± 1.03. The mean gestational age at delivery of the participants is 35.94 ± 4.48 weeks, 37.69 ± 0.85 weeks in planned cases, and 32.33 ± 6.41 weeks in emergency cases. Five of the cases were multiple pregnancies, and all of these cases were delivered in an emergency state. The mean gestational age at birth of twin pregnancies is 32.80 ± 1.92 weeks (ranging between 30 and 35 weeks). Four (1.63%) of all cases had fetal growth restriction, four (1.63%) had premature rupture of the membranes (PROMs), three (1.22%) had gestational hypertensive disorders, three (1.22%) had polyhydramnios, and one (0.41%) had gestational diabetes mellitus.

A total of 80 (32.65%) patients underwent emergency surgery before the planned period, and 11 (4.49%) of them lost their pregnancy before 23 weeks of gestation. The most common causes of preterm delivery in 80 emergency patients were massive vaginal bleeding (*n* = 44, 55.0%), preterm labor (*n* = 13, 16.25%), intra-abdominal hemorrhage (*n* = 6, 7.5%), PROM (*n* = 5, 6.25%), in utero fetal demise (*n* = 3, 3.75%), fetal distress (*n* = 3, 3.75%), multiple fetal anomalies (*n* = 2, 2.50%), severe preeclampsia and HELLP syndrome in patients (*n* = 2, 2.50%), and oligohydramnios (*n* = 2, 2.50%), respectively. Three or more repeated cesarean sections were found in 180 (73.46%) patients. All patients underwent Pfannenstiel skin incision followed by a transverse incision to the upper border of the placenta, and 94.69% (*n* = 232) were operated on under spinal anesthesia.

Preoperative and postoperative mean Hb values, duration of surgery, EBL and amount of transfused erythrocytes per patient, and the length of hospital stay of all patients were presented in Table 2. The mean EBL of the total study cohort was 606 mL. A total of 56 (22.8%) patients received a total of 128 units of RBC transfusion, with an average of 0.52 units per patient (0.36 units per patient in planned cases and 0.85 units per patient in emergency cases). High-dose iron supplementation was administered in the antenatal period to increase the blood values of the anemic patients in our follow-up.

In this study, no cases were indicated of cesarean hysterectomy in surgery due to uncontrolled massive bleeding and secondary hysterectomy due to postpartum intra-abdominal infection and postpartum hemorrhage failed medical treatment. Also, we did not perform hypogastric artery ligation in surgery. The mean operative time was 54.44 ± 11.37 (30–90) min in all patients. Postoperatively, three (1.22%) patients needed ICU; of these, one had HELLP syndrome and two had massive intra-abdominal bleeding. The average length of hospital stay was 1.71 ± 1.30 (1–9) days in all patients, 1.52 ± 1.06 (1–9) days in planned patients, and 2.08 ± 1.64 (1–9) days in emergency patients.

Six patients (2.44%) with placenta percreta had spontaneous massive intra-abdominal bleeding in the antepartum, and one of them (0.41%) had bladder bleeding. Intraoperative bladder injury complications developed in four (1.63%) patients and were primarily repaired. A patient with spontaneous uterine and bladder perforation developed a vesicouterine fistula three months later and was repaired primarily by laparotomy (Table 3).

## 4. Discussion

In the current study, we described our simplified conservative surgery approach and presented the outcomes of our conservative surgical technique in PP patients with PAS disorders. We think that this technique will significantly reduce maternal morbidity and mortality while preserving fertility. This technique provides many benefits such as fertility preservation, less blood loss, and shorter operation time, length of hospital stay, and recovery time.

Four different conservative surgical treatment methods and interventional radiological methods for PAS disorders are described in the FIGO guideline [20]. However, when these methods are compared with our technique, it is seen that none of them is a completely conservative and practical method. In a multicenter retrospective study of 167 cases of PAS disorder that left the placenta in situ, the success rate of uterine preservation was 78% and associated with severe maternal morbidities. Also, maternal death was reported in 10 (6%) cases [26]. In our case series, we preserve the uterus in all patients with a low rate of maternal morbidity. Moreover, no maternal mortality was detected in our study. It has also been reported that ligation and embolization of pelvic vessels caused necrosis of the pelvic organs [27]. In our series, none of the patients underwent pelvic vascular ligation and radiological intervention.

Although there is no consensus on the time of delivery, guidelines recommend scheduled delivery between 34 and 37 weeks in PAS cases. The general approach is to perform a cesarean section after 34 weeks of gestation and to reduce emergency risks [22, 28]. The median gestational age in the planned surgery patients in our study was 37 weeks. As known, scheduling the delivery time at the later weeks of gestation might increase the risk of antenatal bleeding but allows fetal growth and reduces the need for a neonatal ICU admission.

As known, spinal anesthesia is recommended for cesarean delivery to minimize failed intubation and pulmonary aspiration in pregnant women. Also, Apgar scores at 1 and 5 min are lower for general anesthesia (GA) compared with spinal anesthesia [29]. Even though GA should be preferred for severe hemorrhage, several surgeons advocate GA as the primary procedure for PAS cases, stating the high probability of conversion to GA. However, in a recent study, two-thirds of the authors reported a preference for regional anesthesia in PAS cases (followed by conversion to GA if necessary) [30]. In our study, 94.69% of the patients were operated on under spinal anesthesia due to the above-mentioned advantages. The anesthesiologist, who is a member of the multidisciplinary approach, was always ready in all cases for the conversion to GA in necessary conditions.

The decision regarding abdominal and uterine incision sites requires careful consideration in PAS cases. Both the FIGO and the Society of Obstetricians and Gynaecologists of Canada (SOGC) guidelines advise midline skin incision adequately high to provide a hysterotomy above the superior placental margin in PAS cases with major anterior previa. The American College of Obstetricians and Gynecologists (ACOG)/Society for Maternal-Fetal Medicine (SMFM) guidelines indicated that due to the absence of comparative studies, the selection of skin incision is pending the surgeon's choice [1, 31, 32]. However, many surgeons performed vertical midline skin incisions for better access and visualization for the surgery of PAS cases [32]. A midline subumbilical skin incision has the advantages of facilitating access to intra-abdominal adhesions, performing a peripartum hysterectomy more easily if required, and protecting against unintentional bladder injury if the bladder is drawn up [33]. However, a midline incision is correlated with a high rate of postoperative morbidity and might be more cosmetically unsatisfactory than a transverse incision [1, 31, 32]. An upper segment classical uterine incision might hide the placental separation degree causing postponement of blood loss control and challenge in accessing retracted myometrium to suture the tissue compelling another transverse incision to be performed for better visualization. Recently, Mohamed Siraj, Tan, and Wright advocated entrance to the abdomen through a pre-existing transverse skin incision followed by a transverse uterine incision above the uterovesical fold level. They indicated that the single uterine transverse incision through the previous scar has the advantage of providing the placenta to be delivered under direct vision from its attachment on the posterior uterine wall first, followed by removal from the neovascularized anterior wall while tracing the boundary of the sheared posterior myometrial defect before repair. This incision moderates the initial severe blood loss and prevents bladder dissection, thus reducing the rate of bladder injury and bleeding risk of bridging vessels which run over the isthmocele [33]. Also, some other studies employed transverse skin incision and after that a transverse uterine incision in cases with PAS [34, 35]. Similarly, in our study, all patients were operated on with a Pfannenstiel incision followed by a transverse incision to the upper border of the placenta to enter the uterus.

In FIGO guidelines, maternal morbidity and mortality related to PAS surgery have been reported concerning conservative and nonconservative methods in PAS patients [15, 20]. Alanwar et al. reported that the urinary tract injury incidence during cesarean section with PAS was 21.7%, and most of them were bladder injury (11.7%). Preoperatively, they detected approximately 26.9% of PAS cases with bladder invasion according to sonography findings [36]. In our study, a total of nine (3.67%) patients developed intraoperative and postoperative surgical complications and six (2.45%) of them were related to the bladder. We consider that the low rate of urinary tract injury in our study was due to the low rate of bladder invasion cases. Recent studies indicated that preoperative utilization of ureteral catheters in PAS cases does not reduce the risk of urinary system injury [37, 38]. Accordingly, we did not perform double-J catheter insertion before laparotomy to avoid ureteral injury in this study.

In placenta percreta cases, intra-abdominal bleeding may rarely occur even on an unscarred uterus as a result of rupture of placental vessels or lacunar structures, or uterine rupture, and causes acute abdomen. Bladder perforation is extremely rare in cases of placenta percreta [39, 40]. In our study, antenatal spontaneous intra-abdominal bleeding occurred in six patients (2.44%) with placenta percreta, and these were operated under emergency conditions. Based on our clinical experience, patients with large lacunae in the placenta are at higher risk of massive vaginal or intra-abdominal bleeding. In a Canadian institutional experience study, it was found that the mean EBL was 1416 ± 699 mL, the mean duration of surgery was 112 ± 49 min, and the mean hospital stay was 5.2 days. In a single-center study involving 47 invasive placental patients, the mean time duration of surgery was 260 ± 68 min and EBL was 1200 (500–7000) mL in the presence of a multidisciplinary team, while in the absence of a multidisciplinary team, it was 204 ± 55 min and 2500 mL (300–10,000 mL) [41]. In our study, approximately 606 mL of EBL per patient was detected in all patients, the mean time duration of surgery was 54.44 ± 11.37 min, and the mean length of hospital stay was 1.71 ± 1.30 days. In our study, the duration of surgery is lower than in the literature. The main reason for the low duration of surgery is that we define surgery duration as the time interval from skin incision to closure in minutes and we did not calculate this duration from the start of anesthesia. Also, we did not perform a vesicouterine dissection and hemostatic procedures of vascular proliferation before uterine incision or resection of myometrial tissue, which significantly caused the prolongation of surgery. In some countries, discharge the day following a cesarean section without any complication has become an accepted procedure in recent years. Studies reported that discharge within 28 hours can be performed after a cesarean section without compromising the parents' postnatal sense of security [42]. Thus, we performed an early discharge from the hospital of cases without complication.

Nieto-Calvache et al. reported that it is not possible to perform conservative surgery in PAS cases with low parametrial invasion or severe vesicouterine fibrosis [43]. Similarly, Palacios-Jaraquemada et al. indicated that lower PAS parametrial involvement is associated with severe hemorrhage and elevated maternal morbidity [44]. Also, a multidisciplinary team, the availability of blood banks, and ICUs are potentially necessary for cases who desire conservative uterine surgery [45]. In our study, we did not perform a cesarean hysterectomy in any of the cases. In PAS cases, we adopted a multidisciplinary approach including an expert obstetrician and surgeon for PAS, anesthetist, and pediatrician. This team encourages us to perform conservative uterine surgery in PAS cases. Also, we exclude cases with suspected invasion below the bladder trigone and lower parametrial invasion. We consider that this elimination strategy increases the success rates of our surgery.

The main strength of this study is that all PAS cases included in this investigation were diagnosed, operated on, and managed by the same surgeon based on the described conservative surgical procedure. The antenatal diagnosis of PAS cases included in this study was surgically confirmed. Cases without intrasurgical PAS diagnosis were excluded before performing data analysis.

The main limitation of this study is its retrospective nature. We calculated the EBL with a formula and did not quantify it with blood-soaked items. Therefore, the amount of blood loss may have been evaluated with lower accuracy. Also, we did not classify the PAS cases into subtypes as accreta, increta, and percreta, which might introduce another limitation since managing the percreta type is more difficult than others. The lack of intraoperative classification or histologic confirmation limits more definitive conclusions. A probable limitation of this procedure may arise when the placenta invades the lower parametrium or below the bladder trigone due to the increased catastrophic hemorrhage risk. The acquisition of the surgical skills was done by an experienced obstetrician assisting several surgeries before becoming the leader of the team, to provide the reproducibility of the surgical procedure. Therefore, background and surgical skills have enhanced substantially through the years, and the ongoing high rate of success is strongly associated with the existence of a multidisciplinary team of an experienced surgical team, radiologists, neonatologists, anesthetists, and hematologists with the support of a tertiary care center. Therefore, conservative surgical management in PAS cases is better not performed in inadequate healthcare facilities [46]. Also, we did not use a control group to compare our surgical outcomes. Future prospective studies are needed to further evaluate maternal morbidity, surgical success in comparison to other conservative procedures, and the subsequent fertility outcomes.

The surgical management should be carefully considered where applicable, especially in low-resource settings. PAS cases should be managed in a tertiary referral hospital, a consultant-led hospital, or a level-three hospital that provides a multidisciplinary team consisting of an experienced obstetrician, anesthetist, neonatologist, and hematologist [47, 48]. Recently, Nieto-Calvache et al. reported that after experiencing a brief virtual theoretical training program and being assisted by the telepresence of an expert group during surgery, all the participating surgeons who have experience in surgical PAS management successfully carried out conservative surgery in selected cases. They concluded that utilizing telemedicine in the management of PAS cases might improve the evaluation of provider healthcare in low-middle-income countries through proper transfer and strict treatment [49]. Further studies with a more substantial number of interdisciplinary groups included are necessary to evaluate the current value of this intervention in the clinical outcomes of PAS cases.

## 5. Conclusion

An era of advanced maternal intensive care encourages conservative rather than radical surgical procedures in PAS cases. We recommend this procedure as a novel technique and a robust and safe alternative to peripartum hysterectomy and other conservative surgical management procedures for cases with complete PP accompanied with PAS. This technique preserves the uterus as well as reduces blood loss, and transfusion requirement, and thus maternal morbidity and mortality in PAS cases. The surgical method should be reproducible and generalized; thus, many patients could benefit from the conservative surgery, although one surgeon technique could avoid the bias in the study. However, we recommend that surgeons dealing with this surgery should receive practical training and develop their operative experience and surgical skills before utilizing this procedure.

## Figures and Tables

**Table 1:** Patients' characteristics, mean gestational age, and neonate's weight in patients with PAS disorders, mean ± SD (min–max) or median (IQR1-3).

	**All cases (** **n** = 245**)**	**Planned cases (** **n** = 165**)**	**Emergency cases (** **n** = 80**)**	**p** **value**
Maternal age (years)	32.84 ± 5.02 (22–45)	32.98 ± 4.97 (22–44)	32.56 ± 5.14 (22–45)	0.471
Gravida, *n*	4.45 ± 2.06 (2–13)	4.32 ± 2.00 (2–13)	4.72 ± 2.17 (2–13)	0.153
Parity, *n*	2.74 ± 1.57 (0–11)	2.63 ± 1.51 (0–9)	2.97 ± 1.67 (1–11)	0.081
Previous cesarean sections, *n*	3.20 ± 1.03 (1–6)	3.14 ± 1.07 (1–6)	3.32 ± 0.95 (2–6)	0.167
Gestational age at birth (weeks)	33 (29–37)	37 (35–39)	33 (29–36)	**< 0.001** ^∗^
Neonate's birth weight (g)	2937 (2522–3200)	3090 (2865–3300)	2300 (1370–2280)	**< 0.001** ^∗^

*Note:* All other *p* values are from the Student *t*-test. Values in bold indicate statistical significance at the *p* < 0.05 level.

^*^Refers to the Mann–Whitney *U* test.

**Table 2:** Relative characteristics of PAS surgery.

	**All cases (** **n** = 245**)**	**Planned cases (** **n** = 165**)**	**Emergency cases (** **n** = 80**)**	**p** **value**
Preoperative Hb (g)	11.59 ± 1.46 (5.40–14.68)	11.83 ± 1.23 (8.50–14.50)	11.08 ± 1.74 (5.40–14.69)	0.141
Postoperative Hb (g)	10.09 ± 1.41 (6.00–13.60)	10.22 ± 1.38 (6.00–13.60)	9.82 ± 1.44 (6.30–12.80)	**0.049**
Duration of surgery (min)	54.44 ± 11.37 (30–90)	53.48 ± 10.82 (30–90)	56.43 ± 12.25 (30–90)	0.078
Length of hospital stay (days)	1.71 ± 1.30 (1–9)	1.52 ± 1.06 (1–9)	2.08 ± 1.64 (1–9)	**0.002**
Transfused RBCs (units/patient)	0 (0–1)	0 (0–1)	1 (0–1)	0.119^∗∗^
RBC transfused patients, *n* (%)	56 (22.85%)	33 (20%)	23 (28.75%)	0.092^∗^
Estimated blood loss/patient (mL)	606	592	633	0.923

*Note:* Data were presented as mean ± SD (min–max) or *n* (%). All other *p* values are from the Student *t*-test. Values in bold indicate statistical significance at the *p* < 0.05 level.

^*^Refers to chi-square test.

^**^Refers to the Mann–Whitney *U* test.

**Table 3:** Antepartum, intrapartum, and postpartum nonsurgical and surgical complications in patients with PAS disorders.

**Nonsurgical and surgical complications**	**n**	**%**
Antepartum nonsurgical spontaneous complications	6	2.44
Intra-abdominal and bladder hemorrhage	6	2.44
Intrapartum and postpartum surgical complications	9	3.67
Bladder injury	3	1.22
Bladder injury and subsequent bladder atony	1	0.41
Anterior cervical wall and vaginal fornix perforation	1	0.41
Uterine atony and relaparotomy (within 2 hours)	1	0.41
Perforation of the bladder to the abdomen (postpartum fifth day)	1	0.41
Vesicouterine fistula (postpartum third month)	1	0.41
Intestinal serosa injury	1	0.41

## Data Availability

Data will be made available on request from the authors.
